# Squalamine and Aminosterol Mimics Inhibit the Peptidoglycan Glycosyltransferase Activity of PBP1b

**DOI:** 10.3390/antibiotics9070373

**Published:** 2020-07-02

**Authors:** Adrien Boes, Jean Michel Brunel, Adeline Derouaux, Frédéric Kerff, Ahmed Bouhss, Thierry Touze, Eefjan Breukink, Mohammed Terrak

**Affiliations:** 1 InBioS-Centre d’Ingénierie des Protéines, Liège University, 4000 Liège, Belgium; adrien.boes@doct.uliege.be (A.B.); a.derouaux@xpress-biologics.com (A.D.); fkerff@uliege.be (F.K.); 2 UMR_MD1, U-1261, Aix Marseille Univ, INSERM, SSA, MCT, 13385 Marseille, France; bruneljm@yahoo.fr; 3 Institute for Integrative Biology of the Cell (I2BC), Université Paris-Saclay, 91198 Gif-sur-Yvette, France; ahmed.bouhss@univ-evry.fr (A.B.); thierry.touze@u-psud.fr (T.T.); 4 Laboratoire Structure-Activite des Biomolecules Normales et Pathologiques (SABNP), Univ Evry, INSERM U1204, Universite Paris-Saclay, 91025 Evry, France; 5 Membrane Biochemistry and Biophysics, Department of Chemistry, Faculty of Science, Utrecht University, 3584 CH Utrecht, The Netherlands; E.J.Breukink@uu.nl

**Keywords:** peptidoglycan, Lipid II, PBP1b, squalamine, cationic aminosterols

## Abstract

Peptidoglycan (PG) is an essential polymer of the bacterial cell wall and a major antibacterial target. Its synthesis requires glycosyltransferase (GTase) and transpeptidase enzymes that, respectively, catalyze glycan chain elongation and their cross-linking to form the protective sacculus of the bacterial cell. The GTase domain of bifunctional penicillin-binding proteins (PBPs) of class A, such as *Escherichia coli* PBP1b, belong to the GTase 51 family. These enzymes play an essential role in PG synthesis, and their specific inhibition by moenomycin was shown to lead to bacterial cell death. In this work, we report that the aminosterol squalamine and mimic compounds present an unexpected mode of action consisting in the inhibition of the GTase activity of the model enzyme PBP1b. In addition, selected compounds were able to specifically displace the lipid II from the active site in a fluorescence anisotropy assay, suggesting that they act as competitive inhibitors.

## 1. Introduction

Peptidoglycan (PG) is an essential polymer of the bacterial cell wall that surrounds the cytoplasmic membrane, determines the cell shape and protects the cell from lysis. Its metabolism is a major antibacterial target. The PG structure consists of glycan strands made of alternating β-1,4-linked *N*-acetylglucosamine (GlcNAc) and *N*-acetylmuramic acid (MurNAc) residues cross-linked by peptides [1]. It is assembled using the lipid II precursor (undecaprenyl-pyrophosphoryl-MurNAc-(pentapeptide)-GlcNAc) by the glycosyltransferases (GTases) activities of the class A penicillin-binding proteins (aPBPs) and SEDS (shape, elongation, division, and sporulation) proteins and cross-linked by the transpeptidases (TPases) activities of aPBPs and class B PBPs (bPBPs) [2,3,4]. The inhibition of each one of those activities leads to cell death [5].

The GTase domain of bifunctional class A PBPs, such as *Escherichia coli* PBP1b, belong to the GTase 51 family. The enzymatic cavity of these domains can be divided into two substrate binding sites: a donor site for the lipid-bound growing glycan chain, and an acceptor site for lipid II [6]. Moenomycin A is the only known natural product that specifically binds to the GTase 51 donor site and competitively inhibits PG synthesis [7]. It is a potent antibiotic that is not used in human therapy but was used in animal feedstock for decades without reported resistance [8].

**Figure 1:**
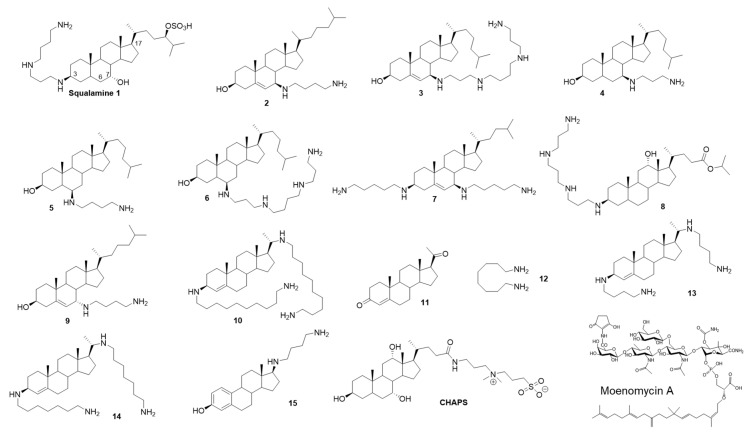
Structures of the aminosterol compounds used in this study and moenomycin A.

Squalamine is a cationic aminosterol antibiotic, isolated from the dogfish shark (*Squalus acanthias*) [9] that exhibits a broad-spectrum antimicrobial activity against Gram-positive and Gram-negative bacteria (MIC for *S. aureus* and *E. coli* ~ 3 µg/mL) and potentiates the activity of several antibiotic classes at subinhibitory concentrations, including against resistant bacteria [10]. Its structure is composed of a steroid linked to a polyamino spermidine moiety (Figure 1), which taken independently exhibit considerably low antibiotic activity and are both required for the observed antimicrobial activity of squalamine [9]. 

In the last decade, numerous aminosterol derivatives of squalamine have been synthesized and characterized [11,12,13,14,15]. Their mode of action was proposed to act by disrupting the outer membrane integrity of Gram-negative bacteria and by depolarizing the cytoplasmic membrane of Gram-positive bacteria [16,17]. Some of them display good membrane selectivity with high activity on bacterial membrane and low hemolytic activity, which make them promising candidates as new antibiotics or as adjuvants to sensitize resistant bacteria to well-known antibiotics [16,17]. 

Herein, we will demonstrate that squalamine and other related aminosterol present an unexpected additional mode of action, consisting of the inhibition of a bacterial PG polymerase. By using in vitro activity assays based on the processing of lipid II substrate into PG polymers by *E. coli* PBP1b, we found that squalamine and six other aminosterol analogs inhibit the GTase activity of this enzyme. In addition, the selected compounds were able to displace the lipid II from the active site in a fluorescence anisotropy assay indicating that they act as competitive inhibitors. 

## 2. Results

### 2.1. Squalamine Inhibits the GTase Activity of PBP1b

**Figure 2:**
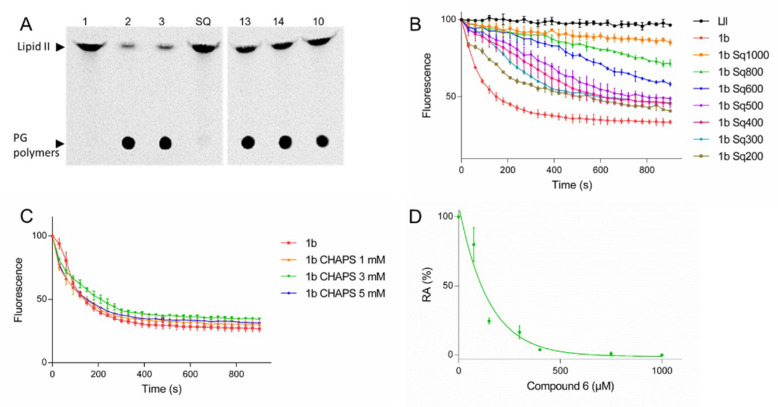
Inhibition of the GTase activity of PBP1b by squalamine and analogs. (**A**) TLC analysis of PG polymerase activity of PBP1b using radioactive lipid II as substrate, in the presence of squalamine and aminosterol analogs. Lanes: 1, lipid II incubated without enzyme; 2–3, lipid II incubated with 100 nM PBP1b with (2) or without (3) addition of methanol (because some compounds tested were solubilized in methanol); and SQ, 13, 14, and 10 depict the reaction incubated in the presence of 0.8 mM squalamine, or compounds **13**, **14** and **10**, respectively. (**B**) Inhibition of PBP1b GTase activity using increasing concentration of squalamine (200–1000 µM) monitored by dansyl-lipid II based continuous fluorescence assay. (**C**) control experiment with CHAPS (1–5 mM). (**D**) a representative experiment of the RA in function of compound concentrations used for the determination of IC_50_ values. The error bars represent the mean values ± s.d. of triplicate experiments.

The crystal structure of the GTase domain of the class A PBP1a from *Aquifex aeolicus* presents a steroidal CHAPS detergent molecule bound to a hydrophobic patch adjacent to the active site [18]. Its side chain was suggested to mimic and partly overlap with diphospholipid chain of the lipid II substrate in the acceptor site. We noticed that the aminosterol antibiotic squalamine and CHAPS have some structural feature in common, consisting essentially of a sterane core (Figure 1). We were curious to know if squalamine could affect the PG polymerase activity of *E. coli* PBP1b. Surprisingly, by using radioactive lipid II as substrate in an endpoint TLC assay we found that 0.8 mM squalamine was able to almost completely inhibit the GTase activity of PBP1b (1.1 ± 0.6% residual activity (RA)), whereas the aminosterol analogs **10**, **13** and **14** were inactive (Figure 2A, Table 1). Variable concentrations of squalamine were then tested using both an endpoint TLC assay and a continuous fluorescence assay that monitor the consumption of dansyl-lipid II substrate over time. The inhibition result shows concentrations-dependent profile and the efficacy of squalamine was rather modest in the high µmolar range (IC_50_ value of 291 ± 26 µM) (Figure 2B, Table 1). When the zwitterionic CHAPS detergent was tested using the same assay at high concentrations and below the critical micelle concentration (CMC 6–10 mM) value, no inhibition of PBP1b GTase activity was observed up to 5 mM (Figure 2C). In addition, steroid progesterone **11** or octanediamine **12** tested alone do not inhibit the GTase activity of PBP1b (Figure 1, Table 1). 

### 2.2. Inhibition of the GTase Activity of PBP1b by Aminosterol Mimics of Squalamine

Based on these encouraging results we have analyzed twelve additional aminosterol compounds with one or more substitutions at C-3, C-6, C-7 and C-17 positions of the sterane rings. To determine the relative activity of the compounds, they were tested at 400 and 800 µM and ranked according to their effect on the residual activity (RA) of the enzyme. Six compounds were found to inhibit the GTase activity of PBP1b with residual activity ranging from 1.2 to 39% and from 0.3 to 8.5% when using compound concentrations of 400 and 800 µM, respectively (Figure 1, Table 1). 

The active compounds were further investigated to determine the IC_50_ values (Table 1). Compounds 2 and 6 were found to be the best ones with IC_50_ values of 114 ± 6 µM and 111 ± 6 µM, respectively, about three times more efficient than squalamine. Interestingly, compound **9**, a stereoisomer of compound **2** at the C-7 position, was almost inactive (RA 98 % at 800 µM). All active compounds (except for squalamine) contain a nonpolar moiety in C-17 and a polar group at position C-3, C-6 or C-7. These results, together with the absence of activity of different analogues (**10**, **13**, **14** and **15**), indicate that the observed inhibitions of the GTase activity of PBP1b by squalamine and analogs are specific and exclude the possibility of promiscuous effect of these series of compounds.

### 2.3. Mode of Action of Aminosterol Compounds

**Figure 3:**
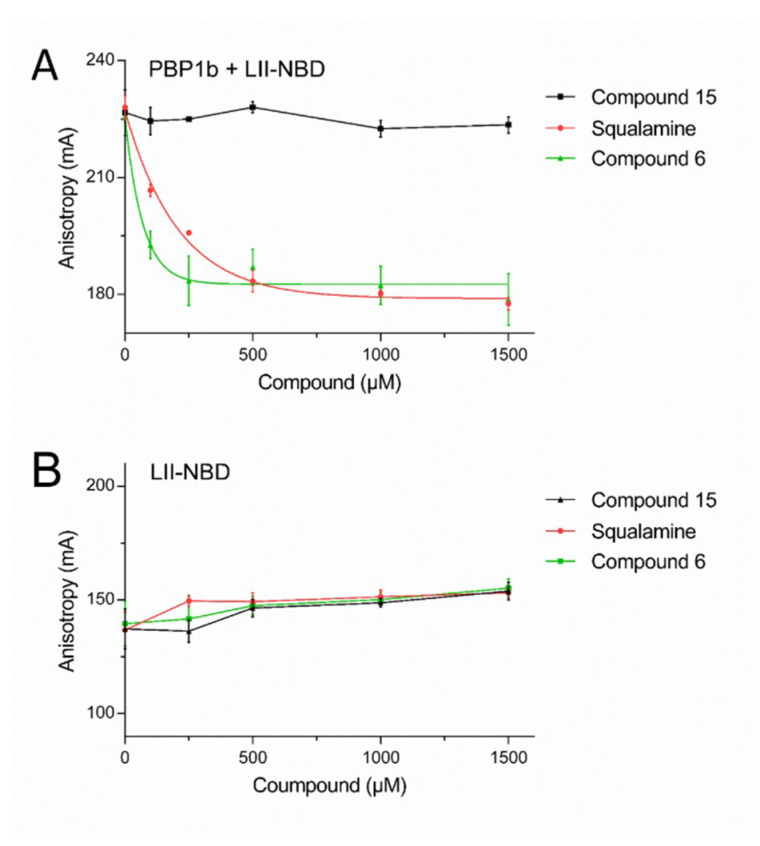
Fluorescence anisotropy assay using the NBD-lipid II as a probe. FA (in mA units) is plotted as a function of compound concentrations. The error bars represent the FA values as mean ± s.d. of triplicate experiments. (**A**) Competition of squalamine and compound **6** for PBP1b/NBD-lipid II binding induce a decrease of the FA of the probe while the inactive compound **15** does not affect the FA. (**B**) Measurements of direct binding between the probe and the compounds show no significant variation of FA, indicating absence of interactions.

The inhibition of the GTase activity can be achieved either by compounds that bind to the active site of the enzyme (e.g., moenomycin A) or through binding to the lipid II substrate (e.g., nisin, ramoplanin). To distinguish between these two mechanisms, we further characterized the mode of action of squalamine, the best inhibitory compound (**6**) and an inactive compound (**15**) by studying their interaction with PBP1b and lipid II, using a recently developed fluorescence anisotropy assay based on a fluorescent lipid II as a probe [19]. The assay was previously validated for direct interaction of the probe with PBP1b and with the antibiotics nisin and ramoplanin (FA increase upon binding), as well as the ability of unlabeled-lipid II, nisin or ramoplanin to displace the probe from the enzyme active site (FA decreases in the presence of competitive compounds) [19]. While no significant interaction was observed between fluorescent lipid II and squalamine or compounds **6** and **15** (Figure 3B), the active compounds (squalamine and **6**) were able to compete with lipid II for binding to PBP1b, whereas the inactive compound **15** had no effect (Figure 3A). These results clearly indicate that the active aminosterol compounds directly interact with the GTase active site of PBP1b. All together the data reveal that squalamine and active analogs have multiple antibacterial modes of action, in addition to their potent membranes damaging activities, they also have modest cell wall synthesis inhibition activity. 

## 3. Discussion

The prevalence of antibacterial resistance is increasing and new solutions should be developed urgently to deal with the resistant pathogens. The GTase activity of bifunctional PBPs is essential for bacterial viability and constitute a proven antibacterial target [20,21]. However, only one natural product antibiotic, moenomycin, that specifically binds to the active site of these enzymes was discovered more than 50 years ago [7,22], and therefore finding a new chemical scaffold able to bind and inhibit this class of enzymes is of great interest. In this regard, the discovery of the natural product squalamine, and synthetic analogues, that inhibit the GTase activity of the model class A PBP1b enzyme, from *E. coli* opens new perspectives for the development of a new class of GTase inhibitors. The identification of aminosterol derivatives with improved activity against the GTase, while maintaining some specific bacterial membranes damaging activity, could result in potent antibacterial agents, particularly against resistant Gram-negative bacteria.

Interestingly, FA experiments indicate that these compounds are competitive inhibitors able to displace the lipid II substrate from the GTase active site, but in contrast to moenomycin which binds only to the donor site and shows an increase in FA upon cooperative binding with lipid II substrate to PBP1b [19,23], the titration of the PBP1b-probe complex by aminosterol compounds induces a decrease in FA signal, similar to that observed in the presence of unlabeled lipid II, indicating that they prevent lipid II probe binding to both donor and acceptor sites. 

**Figure 4:**
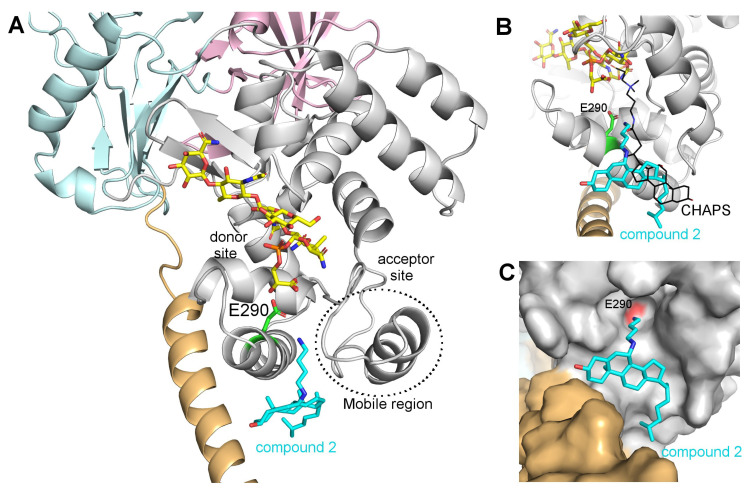
Model of the potential binding mode of compound **2** on the GTase domain of PBP1b (PDB code 5HLB). (**A**) Cartoon representation of PBP1b, with the TGase domain in grey, the trans-membrane segment in pale orange, the UB2H domain in pink and the TPase domain in blue. Moenomycin A and compound **2** are represented as yellow and cyan sticks respectively. The catalytic E290 is shown as green sticks. (**B**) Same as (**A**) with a rotation of approximately 90° and a CHAPS molecule represent as back lines. (**C**) Same as (**B**) with PBP1b represented as a surface. The red patch indicates the position of the accessible Oε from E290.

A model of the potential binding mode of compound **2** was obtained by analogy with the *A. aeolicus* PBP1a structure in the presence of CHAPS (Figure 4A–C) [18]. The steroidal part of compound **2** binds on an equivalent hydrophobic patch on PBP1b, but it is rotated and slightly shifted so that the polar chain follows the same path as the CHAPS molecule. Interestingly, the amine extremity of the polar chain is then located in close proximity (3.3 Å) of the catalytic E290 residue. This binding mode would be conserved for active compound **3** to **7** that have their polar chain at the same position as compound **2** or on the adjacent carbon and longer polar chains would reach farther into the active site. For squalamine, it is not clear if the steroidal core would be shifted or the longer polar chain would follow a different path to reach E290. 

As indicated above, these compounds seem to compete with the binding of lipid II in both the donor and acceptor sites. It is therefore not clear if a second binding site exists in the acceptor site or if the binding of aminosterol compounds to the donor site induces a conformational change (of the mobile region separating the two sites for example) that prevents the binding of lipid II to the acceptor site. The structure of PBP1b in complex with one of these compounds is therefore required to validate this hypothesis as well as the proposed binding mode.

Moenomycin has an excellent antibacterial potency and no reported resistance despite extensive use in animal feeds, but it is not used in human therapy because of its undesirable physical properties that result in poor bioavailability and long serum half-life, probably due to its lipidic C25 moenocinol moiety [8,24,25]. This lipid tail is necessary for moenomycin activity and its replacement by an alternative one could improve the properties of the resulting molecule. The C25 lipid tail of moenomycin was not resolved in any crystal structure of GTase-moenomycin complexes, indicating that it does not adopt an ordered conformation [6,26]. In contrast, CHAPS was well defined in the structure of the GTase domain of PBP1a from *A. aeolicus* [18] suggesting that binding of the cyclic steroid-substituted compounds would provide a defined structure and facilitate structure-based optimization of compounds.

Most of GTase inhibitors (moenomycin and synthetics compounds) are inactive against Gram-negative bacteria [21]. On the other hand, aminosterols have the capacity to cause lesions in the outer membrane and either kill, or sensitize, Gram-negative resistant strains to common antibiotics. Therefore, it is tempting to speculate that aminosterol compounds may be useful substitutes for the lipid moenocinol part of moenomycin (or to the lipid tail of lipid II analogs [27]) to generate useful new hybrid compounds, consisting of the carbohydrate and phosphoglycerate moieties or the minimal moenomycin pharmacophore (disaccharide (EF)-phosphoglycerate [21]) and an aminosterol tail (Figure 4). Such compounds would have two functions: (i) to replace the lipid tail of moenomycin derivatives while maintaining their binding to the GTase active site; and (ii) to allow these compounds to cross the outer membrane (via the polyamine cationic moiety) and gain effectiveness against Gram-negative pathogens. 

## 4. Material and Methods

### 4.1. Reagents and Protein

Labelled lipid II [N-acetylglucosaminyl-N-acetylmuramoyl (L-Ala-γ-DGlu-(L)-Lys-(L)-D-Ala-D-Ala)-pyrophosphate-undecaprenol] variants were prepared as previously described: Dansyl-lipid II [28], NBD-lipid II [29], [^14^C]-Lipid II (0.06 µCi nmol^−1^) [30]. 

PBP1bγ(M46-N844) was purified as previously described [31]. 

### 4.2. Compounds Synthesis 

Methanol, ethyl acetate and dichloromethane were purchased from SDS and used without further purification. Column chromatography was performed on SDS silica gel (70–230 mesh). NMR spectra were recorded in MeOD on a Bruker AC 300 spectrometer. Mass spectroscopy analysis have been performed by the Spectropole of Aix-Marseille Université (Marseille). The purity of the compounds was checked by analytical HPLC (C18 column, eluent CH_3_CN-water-TFA (90:10:0.025, v/v/v), 0.5–1 mL/Min) with PDA detector spanning from 210 nm to 310 nm. All compounds possessed purity above 95%, as determined by analytical LCMS Agilent.

Octanediamine **11** and progesterone **12** were purchased from Sigma-Aldrich (Saint Quentin Falavier, France). Squalamine **1** was prepared according to reported procedures [32,33]. Derivatives **2**, **3** and **9** were prepared according [34]. Derivative **4** was prepared according [35]. Derivatives **10**, **13** and **14** were prepared according [13]. Claramine A1 **8** was prepared according [36]. 

#### 4.2.1. General Procedure for the Titanium–Mediated Reductive Amination Reaction

6β-(spermine)-cholestan-3β-ol **6**: A mixture of 6-ketocholestanol (157 mg, 0.39 mmol), titanium(IV) isopropoxide (573 µL, 2.02 mmol) and spermine (202 mg, 1 mmol) were placed under argon in a two-necked round flask. 5 mL of absolute methanol was then added, and the mixture stirred at room temperature for 12 h. Sodium borohydride (38 mg, 1 mmol) was added in portions at -78 ºC and the stirring was maintained for an additional 2 h. The reaction was then quenched by adding water (1 mL) and the mixture stirred 20 min at 20 °C. After filtration over a pad of Celite, the combined organic phases were dried over Na_2_SO_4_, concentrated under vacuum. The crude mixture was then purified by flash chromatography to afford the expected amino derivative. 

Purification by column chromatography (silica gel; CH_2_Cl_2_/MeOH/NH_4_OH(32%), 7:3:1) afforded a pale yellow solid in 45% yield; this compound can be converted subsequently into its hydrochloride salt as white solid –^1^H NMR (300 MHz, CD_3_OD): *δ* = 0.67–0.76 (m, 4H), 0.90–1.17 (m, 24H), 1.20–1.46 (m, 7H), 1.47–1.92 (m, 20H), 2.03–2.07 (m, 1H), 2.50–2.54 (m, 1H), 2.63–2.73 (m, 13H), 3.56–3.63 (m, 1H) –^13^C NMR (75 MHz, CD_3_OD): *δ* = 71.50, 58.96, 56.27, 56.05, 54.78, 49.98, 49.21, 47.99, 47.84, 47.34, 42.62, 40.47, 39.94, 39.49, 39.06, 36.44, 36.14, 35.86, 35.77, 35.63, 33.58, 31.61, 30.45, 28.18, 27.97, 24.37, 23.78, 22.78, 22.52, 21.03, 18.63, 16.30.12.13. C_37_H_72_N_4_O; MS (ESI) m/z = 589.5 [M + H]^+^.

#### 4.2.2. 6β-(1,4-diaminobutane)-cholestan-3β-ol **5**

Purification by column chromatography (silica gel; CH_2_Cl_2_/MeOH/NH_4_OH(32%), 7:3:1) afforded a pale yellow solid in 73% yield; – ^1^H NMR (300 MHz, MeOD): δ = 0.66-3.57 (m, 58H) – ^13^C NMR (75 MHz, MeOD): δ = 71.65, 59.88, 58.54, 56.29, 56.04, 54.75, 48.18, 47.29, 42.71, 42.64, 39.94, 39.50, 39.04, 36.16, 35.78, 35.65, 31.56, 31.03, 30.40, 29.67, 27.99, 25.96, 24.35, 23.81, 22.79, 22.54, 21.05, 18.65, 16.33, 14.09, 12.15. C_31_H_58_N_2_O; MS (ESI) m/z = 475.4 [M + H]^+^.

#### 4.2.3. N1,N1’-((3S,7R,10R,13R,17R)-10,13-dimethyl-17-((R)-5-methylhexan-2-yl)-tetradecahydro-1H-cyclopenta[a]phenanthrene-3,7-diyl)bis(pentane-1,5-diamine) **7**

Synthesis according a similar protocol than for derivative **5** but using 3,7-diketocholestene and 1,5-diaminopentane as starting materials. Purification by column chromatography (silica gel; CH_2_Cl_2_/MeOH/NH_4_OH(32%), 7:3:1) afforded a pale yellow solid in 48% yield; – ^1^H NMR (300 MHz, MeOD): δ = 0.68–0.70 (m, 3H), 0.83–0.87 (m, 7H), 0.91–0.93 (m, 3H), 1.01–1.03 (m, 3H), 1.06–1.08 (m, 2H), 1.09–1.18 (m, 4 H), 1.19–1.25 (m, 4H), 1.27–1.35 (m, 8H), 1.36–1.55 (m, 8H), 1.57–2.03 (m, 13H), 2.31–2.37 (m, 1H), 2.59–2.87 (m, 7H), 2.94–3.01 (m, 2H), 5.36–5.38 (m, 1H). – ^13^C NMR (75 MHz, MeOD): δ = 140.88, 125.41, 55.81, 55.47, 55.07, 52.16, 47.41, 47.13, 47.02, 42.85, 40.71, 40.61, 40.45, 39.50, 39.40, 38.40, 37.52, 37.25, 36.22, 35.70, 32.97, 32.87, 30.36, 30.26, 29.05, 28.87, 28.19, 24.78, 24.24, 24.14, 24.06, 22.66, 21.32, 18.76, 18.65, 12.35. C_36_H_68_N_4_; MS (ESI) m/z = 557.54 [M + H]^+^.

#### 4.2.4. (13S,17S)-17-((4-aminobutyl)amino)-13-methyl-7,8,9,11,12,13,14,15,16,17-decahydro-6H-cyclopenta[a]phenanthren-3-ol **15**

Synthesis according a similar protocol than for derivative **5** but using oestrone and 1,4-butanediamine as starting materials. Purification by column chromatography (silica gel; CH_2_Cl_2_/MeOH/NH_4_OH(32%), 7:3:1) afforded a pale yellow solid in 37% yield; – ^1^H NMR (300 MHz, MeOD): δ = 0.77–1.58 (m, 17H), 2.02–2.50 (m, 3H), 2.66–2.74 (m, 5H), 3.13–3.30 (m, 3H), 3.43–3.63 (m, 2H), 6.50–7.08 (m, 3H), 8.06 (s, 1H). – ^13^C NMR (75 MHz, MeOD): δ = 156.55, 139.17, 132.80, 127.63, 116.65, 114.32, 70.45, 53.94, 45.69, 44.57, 42.29, 40.75, 40.60, 36.24, 31.15, 30.62, 29.13, 28.10, 24.87, 23.05, 20.46, 14.74, 12.77, 12.69. C_22_H_34_N_2_O, M = 342.53 g.mol^−1^, Calc. C 77.15, H 10.1, N 8.18, Exp.C 77.12, H 11.3, N 8.21.

### 4.3. GTase Activity Assays

The continuous fluorescence GTase activity assay of PBP1b was performed using dansyl lipid II as substrate as described [37,38]. 

The end-point assay, based on radiolabeled lipid II ([^14^C]lipid II), was performed in 30 µL in the same conditions as the fluorescence assay using 4 µM [^14^C]lipid II (0.06 µCi/nmol**)** instead of the fluorescent substrate and by omitting the muramidase. The reaction was incubated 15 min at 30 °C, stopped by heating at 80 °C for 10min and the products were separated by thin-layer chromatography (TLC) on silica gel plates in 2-propanol–ammonium hydroxide (25%)–H_2_O (6:3:1; v/v/v). The TLC plates were exposed to a storage phosphor screen (GE Healthcare) for 16 h, and images were revealed using a Typhoon Trio imager and Image Quant TL software (GE Healthcare).

The GTase inhibition assay was carried out by measuring the residual activity (RA) of *E. coli* PBP1b in the presence of 400 and 800 μM compounds. All the assays were repeated at least three times. The IC_50_ values were determined for the active compounds using variable concentrations of inhibitor (0−1000 μM) by fitting the data using GraphPad Prism 6.0 software.

### 4.4. Fluorescent Anisotropy (FA) Binding Assay

FA assay was performed as previously described [19]. Briefly, fixed concentrations of NBD-lipid II (0.33 µM) and PBP1b (~1 µM) in the 25 mM Tris–HCl pH 7.5, NaCl 0.1 M, CHAPS 0.14% *w/v*, were mixed with serial dilutions of the compounds in 384-well plates. Measurement were performed using an Infinite F Plex (Tecan, Männedorf, Switzerland) microplate reader equipped with polarization filters with excitation and emission wavelengths at 485 and 535 nm respectively. All the experiments were performed in triplicates. For direct binding evaluation of the compounds with the probe, the experiments were performed in the same condition in the absence of the protein. FA values in millianisotropy units (mA) were plotted as a function of compound concentrations in µM as previously described [19].

### 4.5. Modeling

The model of the interaction between compound **2** and PBP1b was obtained by manually placing compound **2** based on the structure of *A. aeolicus* PBP1a bound to a CHAPS molecule [18] using the software coot [39]. Compound **2** was positioned so that its steroidal part interacts with the same hydrophobic patch as CHAPS, but slightly shifted so that its polar chain follows the same path towards the active site as the CHAPS chain. An energy minimization procedure of the Yasara software [40] was then applied. This procedure consists in a steepest decent energy minimization followed by a simulated annealing minimization with the Yasara2 forcefield.

## 5. Conclusions

Squalamine and cationic aminosterol analogues are broad-spectrum antibiotics that target bacterial membranes. We now show that some of these compounds have an additional mode of action consisting of the inhibition of the glycosyltransferase activity of PBP1b. This finding opens new perspectives for the development of a new class antibiotics against the GTase, which is considered an important antibacterial target.

## Figures and Tables

**Table 1:** GTase inhibition activities of squalamine and aminosterol analogues.

Compounds	RA % (0.8 mM)	RA % (0.4 mM)	IC_50_ (µM)
**Squalamine (1)**	1.1 ± 0.6	6 ± 2	291 ± 26
**2**	0.3 ± 0.1	1.2 ± 0.3	114 ± 6
**3**	8.5 ± 3.1	16 ± 2	220 ± 10
**4**	4 ± 1	39 ± 2	303 ± 15
**5**	3 ± 1	16 ± 1.4	221 ± 26
**6**	0.8 ± 0.4	3.9 ± 0.1	111 ± 6
**7**	1.5 ± 0.8	12.5 ± 4.4	229 ± 17
**8**	97 ± 0.2	nd	nd
**9**	98 ± 1	nd	nd
**10**	99 ± 0.5	nd	nd
**11**	96 ± 2	nd	nd
**12**	98 ± 1	nd	nd
**13**	100	nd	nd
**14**	100	nd	nd
**15**	105 ± 3	nd	nd

RA = the residual GTase activities values of PBP1b in % of inhibition compared to nontreated control are shown for two compounds concentrations (400 and 800 µM). Both RA and IC_50_ data represent mean values of three independent experiments ± s.d. nd, not determined. Squalamine (**1**) and analogues are in bold numbers (**2–15**).
